# The consequences of sudden fluid shifts on body composition in critically ill patients

**DOI:** 10.1186/cc13794

**Published:** 2014-03-25

**Authors:** Sophie Ismael, Magali Savalle, Claire Trivin, Florence Gillaizeau, Christian D’Auzac, Christophe Faisy

**Affiliations:** 1 Service de Réanimation Médicale, Université Paris Descartes, Hôpital Européen Georges Pompidou, Assistance Publique - Hôpitaux de Paris, Paris, France; 2 Service de Néphrologie-Hémodialyse, Université Paris Descartes, Hôpital Européen Georges Pompidou, Assistance Publique - Hôpitaux de Paris, Paris, France; 3 Unité d’Epidémiologie et de Recherche Clinique, Université Paris Descartes, Hôpital Européen Georges Pompidou, Assistance Publique - Hôpitaux de Paris, Paris, France

## Abstract

**Introduction:**

Estimation of body composition as fat-free mass (FFM) is subjected to many variations caused by injury and stress conditions in the intensive care unit (ICU). Body cell mass (BCM), the metabolically active part of FFM, is reported to be more specifically correlated to changes in nutritional status. Bedside estimation of BCM could help to provide more valuable markers of nutritional status and may promote understanding of metabolic consequences of energy deficit in the ICU patients. We aimed to quantify BCM, water compartments and FFM by methods usable at the bedside for evaluating the impact of sudden and massive fluid shifts on body composition in ICU patients.

**Methods:**

We conducted a prospective experimental study over an 6 month-period in a 18-bed ICU. Body composition of 31 consecutive hemodynamically stable patients requiring acute renal replacement therapy for fluid overload (ultrafiltration ≥5% body weight) was investigated before and after the hemodialysis session. Intra-(ICW) and extracellular (ECW) water volumes were calculated from the raw values of the low- and high-frequency resistances measured by multi-frequency bioelectrical impedance. BCM was assessed by a calculated method recently developed for ICU patients. FFM was derived from BCM and ECW.

**Results:**

Intradialytic weight loss was 3.8 ± 0.8 kg. Percentage changes of ECW (-7.99 ± 4.60%) and of ICW (-7.63 ± 5.11%) were similar, resulting ECW/ICW ratio constant (1.26 ± 0.20). The fall of FFM (-2.24 ± 1.56 kg, -4.43 ± 2.65%) was less pronounced than the decrease of ECW (*P* < 0.001) or ICW (*P* < 0.001). Intradialytic variation of BCM was clinically negligible (-0.38 ± 0.93 kg, -1.56 ± 3.94%) and was significantly less than FFM (*P* < 0.001).

**Conclusions:**

BCM estimation is less driven by sudden massive fluid shifts than FMM. Assessment of BCM should be preferred to FFM when severe hydration disturbances are present in ICU patients.

## Introduction

Nutritional support is based on the assumption that critically ill patients are prone to develop malnutrition. However, nutritional status evaluation in critically ill patients remains an unresolved issue and the lack of reliable reference parameters for energy/calorie intake calculation, when indirect calorimetry is not available or applicable, makes protein-energy malnutrition common in ICU patients whatever their nutritional status at admission [1,2]. This situation is attributable to a combination of reduced caloric intake, hypermetabolism and hypercatabolism in ICU patients. Indeed, the largest negative energy balance, that corresponds to cumulated energy delivered minus cumulated resting energy expenditure (REE), observed in the first days after ICU admission results in a negative gap that is not filled thereafter [3,4]. Adapting caloric intakes to REE has long been considered a minor issue in the first days of ICU hospitalization while energy deficit is now correlated with various complications [5,6]. Patients with major energy deficit are reported to have a longer ICU stay, prolonged mechanical ventilation and are more frequently exposed to nosocomial infections [3-8]. Because of its impact on ICU morbidity, early nutritional assessment might be a major challenge in order to prevent further complications and, therefore, reduce ICU mortality [9]. However, recent studies dealing with the concept of supplemental parenteral to enteral nutrition in critically ill patients have the effect of confusing the debate on the reduction of the energy deficit on morbidity and outcomes in ICU patients [5,6,10].

The further development of clinical tools for assessing protein-energy deficit could help to prove the relevance of the concept of optimal protein-energy intakes in ICU patients. Nutritional markers, such as body mass index (BMI), albuminemia or transferrin, are made unreliable in ICU practice because of systemic inflammation and fluid balance disturbances [11]. Moreover, estimation of body composition as fat-free mass (FFM) is subject to many variations caused by injury and stress conditions in the ICU [12,13]. Body cell mass (BCM), the metabolically active part of FFM, is reported to be more specifically correlated to changes in nutritional status [11,14-16]. Indeed, BCM is closely linked to REE [14]. In addition, a lower BCM/FFM ratio is associated with higher extracellular water/intracellular water (ECW/ICW) [17]. It has been suggested that assessing BCM, BCM/weight or BCM/FFM ratios could help to provide more valuable markers of nutritional status and may promote understanding of the metabolic consequences of energy deficit [11,12]. Because of fluid shifts and poor renal and intestinal function during critical illness, the isotope and tracer dilution methods used for BCM measurements are impractical in critically ill patients, at least in the first or second week after their admission [15,18,19]. For this reason, a calculated method to determine BCM at bedside in critically ill patients has been recently developed [12].

Protein-energy malnutrition in patients on chronic hemodialysis is characterized by decreases of proteins, serum albumin, transferrin and muscle mass caused by losses of plasma and muscle free amino acids during maintenance hemodialysis, chronic inflammation and uremia toxin generation [20-22]. However, protein-energy wasting is difficult to assess in patients on chronic hemodialysis [20]. As FFM is modified, BCM might be changed by chronic hemodialysis [21,22]. Chronic dialysis patients are different from critically ill patients so that the relationship between acute hemodialysis and changes in body composition remains unclear in ICU patients.

Bioelectrical impedance analysis (BIA) is a measure of resistance to the flow of an alternating electrical current through the body. This technique, easy to use, quantifies body water compartments and can indirectly estimate FFM or BCM. BIA has been widely used for many years to assess water compartments at bedside. However, single-frequency BIA is made unusable in the case of short-term water compartments fluctuations and the usual predictive formulas used to determine BCM or FFM are not validated in the case of fluid overload [23-26]. In contrast, multi-frequency BIA has been reported as the best tool to assess ECW and fluid overload at bedside in chronic hemodialysis patients [27,28].

We prospectively quantified fluid dynamics and changes in body composition at bedside during acute hemodialysis in ICU patients by using the raw values of the low- and high-frequency resistances measured by multi-frequency BIA and a BCM calculating method with the aim of assessing the clinical relevance of the current model of BCM calculation in the case of massive fluid disturbance.

## Materials and methods

### Study setting and patient sample

This prospective study was conducted over a six-month period in the medical ICU of our hospital. We evaluated adults who developed acute anuric renal failure during their ICU stay and required renal replacement therapy for fluid overload (ultrafiltration estimated ≥5% body weight before the hemodialysis session). The study was approved by our local Ethics Committee (Comité de Protection des Personnes Ile de France II) and patients or immediate family members gave written informed consent or assent, respectively. Patients were included when their condition was compatible with conventional hemodialysis and met the following steady-state criteria: 1) hemodynamic: no introduction or dose modification of vasoactive drugs during the preceding four hours; 2) respiratory: fraction of inspired oxygen (FiO_2_) <60%, no ventilator adjustments during the preceding four hours, and no signs of hyperventilation (respiratory rate >35/min) or respiratory weakness; and 3) no agitation. Exclusion criteria were pregnancy, administration of diuretics, and any clinical conditions responsible for erroneous calculations of water compartments or BCM as previously described [12]: 1) pacemaker or implanted cardiac defibrillator; 2) amputated limb; 3) orthopedic prosthesis/implants (metal); 4) BMI >40 or <15 kg/m^2^; 4) abnormal body geometry (scoliosis or atrophy); 5) ascites; and 6) skin lesions at the site where the BIA electrode should be placed.

### Anthropometry

Body weight was measured at ICU admission and the day of measurements (immediately before and 30 minutes after the hemodialysis session) by means of an electronic scale (ARJO, Gloucester, UK) with an accuracy of ± 100 g. Using a measuring tape, height was determined immediately before and 30 minutes after the hemodialysis session with the patient lying in the same supine position, as were trunk length (from the upper thigh to the shoulder) and right leg length (from the ankle bone to upper thigh), and the mean circumference of the right leg (mean of thigh, knee, and ankle circumferences) as previously described [29].

### Intra- and extracellular water volumes measurements

All tissues have resistance to the flow of an electric current. Highly conductive tissues contain great quantities of water and conducting electrolytes characterized by a low resistance electrical pathway. Reactance represents the opposition to the flow of electric current caused by capacitance of cell membranes. In fact, the cell membrane could be considered as a layer of non-conductive lipid material sandwiched between two layers of conductive protein molecules that behaves as a capacitor when exposed to an alternating current. Theoretically, the cell membrane represents a permeable barrier separating the ICW and ECW. High-frequency alternating current flows through ICW and ECW whereas low frequency current flows only through ECW because of the resistance due to the capacitance of cell membranes. To assess the water compartments in critically ill patients, we used the multiple-frequency bioimpedance analyzer SFB7 (Impedimed, Eight Mile Plains, Australia), which has a 200-*μ*A alternating current at 4 to 1,000 kHz. The impedance range is 10 to 1,100 Ω with an accuracy of ± 1% (50 to 1,100 Ω) to ± 5% (<50 Ω). The device has a tetra-polar set of leads, which are attached to self-adhesive skin electrodes placed on the right hand (wrist next and dorsal surface, 1 cm proximal to the middle knuckle) and foot (ankle at the level of the protruding bones on the sides of the ankle and at the base of the toes, 1 cm proximal to the joint of the second toe). Before measurements, patients were in a fully supine position, with their arms lying relaxed at their sides but not touching the body and thighs separated. Self-adhesive skin electrodes were placed immediately before hemodialysis for the first set of BIA-measurements and then removed. Thirty minutes after the hemodialysis session, new skin electrodes were placed back in the same position for the second set of BIA-measurements. The resistance R and the reactance Xc were measured directly in Ω at 5 kHz and 1 MHz. Only the ECW is conductive at low frequency (5 kHz), whereas high frequency (1 MHz) allows the electric current to pass through the ECW and ICW. Three consecutive measurements were obtained immediately before and 30 minutes after the hemodialysis session and the R and Xc means were computed. Repeatability of the BIA measurements with this device is excellent in critically ill patients with fluid overload as was shown in a previous study [12]. The impedance Z was calculated as follows: Z = $\sqrt{R^{2}+Xc^{2}}$. Resistance R and impedance Z refer to the opposition of an object to direct and alternating current, respectively. The reactance caused by the resistive effect due to the capacitance produced by tissue interfaces and cell membranes becomes negligible at 5 KHz and 1 MHz and Z becomes equal to R. Accordingly, R_1_ and R_2_ were obtained at low frequency (5 KHz) and high frequency (1 MHz), respectively. We normalized R_1_ and R_2_ by the square of the body height (H) because H^2^/R_1_ is a linear function of ECW and H^2^/R_2_ is correlated with FFM and BCM in healthy adults [12]. Total body water (TBW), ECW and ICW were computed according to the biophysical model developed by De Lorenzo *et al.* and by Matthie (see below) [30,31]. ECW was calculated as follows: ECW = *K*_ECW_ × ${\left(\frac{H^{2}\sqrt{W}}{R_{1}}\right)}^{2/3}$ where H is body height (in cm) and W is weight (in kg). *K*_ECW_ is a factor related to body geometry, density and resistivity as follows: *K*_ECW_ (L) = $\frac{1}{1000}\times {\left(\frac{K_{B}^{2}\times \rho_{\mathrm{ECW}}^{2}}{D}\right)}^{1/3}$ where D is body density (1.05 × 10^-3^ kg/cm^3^, that is 1.05 kg/L), *ρ*_ECW_ is ECW resistivity (40.5 Ω/cm for men and 30 Ω/cm for women), and *K*_B_ is a coefficient accounting for body height-to-limb geometry. *K*_B_ was calculated as follows: *K*_B_ = $\frac{1}{H^{2}}\times \left[\left(\frac{L_{A}}{C_{A}^{2}}+\frac{L_{T}}{C_{T}^{2}}+\frac{L_{L}}{C_{L}^{2}}\right)\times \left(2\times L_{A}\times C_{A}^{2}+L_{T}\times C_{T}^{2}+2\times L_{L}\times C_{L}^{2}\right)\right]$ where *C*_*A*_, *C*_*T*_, and *C*_*L*_ represent segmental arm, trunk and leg circumferences (cm) and *L*_*A*_, *L*_*T*_, and *L*_*L*_ are the lengths of those segments (cm). ICW was calculated as follows: ICW = ECW × $\left\{{\left[\frac{\rho_{\mathrm{TBW}}\times \left(R_{1}+R_{\mathrm{ICW}}\right)}{\rho_{\mathrm{ECW}}\times R_{\mathrm{ICW}}}\right]}^{2/3}-1\right\}$ and *ρ*_TBW_ = *ρ*_ICW_ - (*ρ*_ICW_ - *ρ*_ECW_) × ${\left(\frac{R_{\mathrm{ICW}}}{R_{1}+R_{\mathrm{ICW}}}\right)}^{2/3}$ where *ρ*_TBW_ is total body water resistivity, *ρ*_ECW_ is ECW resistivity, *ρ*_ICW_ is ICW resistivity (273.9 Ω/cm for men and 264.9 Ω/cm for women), R_ICW_ is intracellular resistance (Ω). R_ICW_ was calculated as follows: $R_{\mathrm{ICW}}=\frac{R_{1}\times R_{2}}{R_{1}-R_{2}}$. TBW is the sum of ECW and ICW.

### Body cell mass and fat-free mass calculations

BCM was calculated with the following specific model for the ICU patients [12]: BCM (kg) = 0.266 × height (cm) + 0.287 × mean leg circumference (cm) + 0.305 × Δ weight (kg) – 0.406 × trunk length (cm) – 13.52 where Δ weight represents the weight shift between ICU admission and BCM assessment. FFM could be expressed as the sum of BCM, extracellular fluid (ECF) and extracellular solids (ECS) at the cellular level [12,16,17]: FFM = BCM + ECF + ECS. Assuming ECS = 0.73 × Mo and ECF = ECW/0.98 [32], FFM can be calculated with the following equation: FFM = BCM + (ECW/0.98) + (0.73 × Mo) where Mo (bone mineral) was 2.1 kg as previously measured by dual-energy-X-ray absorptiometry in an earlier group of ICU patients [12]. Accordingly, we calculated the BCM/weight and BCM/FFM ratios, two determinants of nutritional status [11,17].

### Hemodialysis

Renal replacement therapy was performed for three to four hours using the AK200 hemodialysis machine (Gambro SAS, Colombes, France) with the Sureflux FH**-**150 super-permeable cellulose triacetate membrane (Nipro, Osaka, Japan). All patients had a central venous hemodialysis catheter (Arrows, Reading, PA, USA) inserted through the right internal jugular vein. For each patient, the following were recorded: the intradialytic weight loss (UF), the pre- and post-dialysis plasma creatinine and sodium concentrations, the pre- and post-dialysis measured plasma osmolality and the dialysate sodium concentration. The intradialytic sodium gradient (GNa) was calculated as the difference between dialysate and post-dialysis serum sodium concentrations. The intradialytic sodium balance can be estimated from the pre- and post-dialysis plasma sodium concentrations, pre-dialysis TBW and UF as follows [33]: sodium balance (mmol) = Na_post-dialysis_ × (TBW_pre-dialysis_ + UF) - (Na_pre-dialysis_ × TBW_pre-dialysis_)_._

### Patient data

We recorded the following: nutritional status at admission was considered as malnourished when BMI <19 kg/m^2^ or weight <90% ideal body weight [34]; at 24 hours post-ICU admission: simple acute physiology score (SAPS) II; anthropometrics, BIA measurements, calculation of water compartments and BCM, and estimation of FFM were achieved immediately before hemodialysis and 30 minutes after dialysis, thereby limiting bias due to intercompartmental fluid shifts induced by hemodialysis. Enteral nutrition was stopped between the two-point measurements and the volume of perfusion fluids administered was considered negligible. Parenteral nutrition was not used.

### Statistical analysis

Results are expressed as numbers (%) or means ± SD. Statistical analyses were performed by repeated t-test and by Fisher’s exact test or Kruskal-Wallis test for categorical variables (StatView 5.0, SAS Institute, Cary, NC, USA). The Spearman’s correlation coefficient *r* was calculated by using linear regression analysis. Significance was defined as *P* <0.05.

## Results

**Figure 1:**
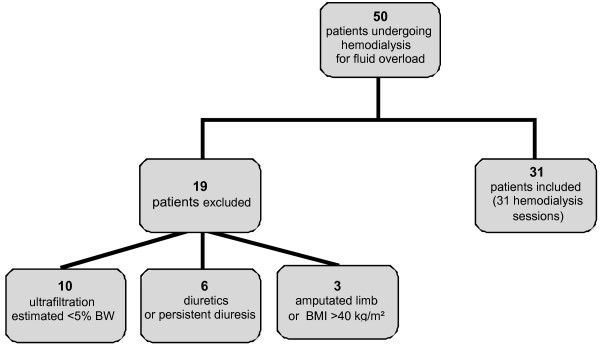
**Study flow chart.** BMI, body mass index; BW, body weight.

Over a six-month period, the sample population consisted of 50 consecutive patients with renal impairment who were undergoing hemodialysis for fluid overload. Nineteen patients met exclusion criteria resulting in 31 patients who were eligible for body composition assessment (Figure 1). Demographic and descriptive data of the 31 patients included in the study are summarized in Table 1.

**Table 1:** Characteristics of the 31 critically ill patients evaluated for fluid dynamics and body-composition assessment

**Characteristics**	**Value**
Demographics and health status	
Age, years (±SD)	62.4 ± 19.5
Male gender, number (%)	19 (61.2)
SAPS II at ICU admission (±SD)	60.0 ± 19.5
Weight at ICU admission, kg (±SD)	64.8 ± 10.5
Height, cm (±SD)	167 ± 11.1
Body mass index at ICU admission, kg/m^2^ (±SD)	23.9 ± 3.8
Malnourished at ICU admission, number (%)	4 (12.9)
ICU stay	
Reason for ICU admission, number (%)	
Septic shock	16 (51.6)
Cardiogenic shock	7 (22.6)
Neurological failure	5 (16.1)
Postoperative complications	3 (9.7)
Day of body composition assessment	
Length of ICU stay before measurements, days (±SD)	7.9 ± 4.7
Mechanical ventilation, number (%)	18 (58.1)
Vasopressors, number (%)	0
Body temperature, °C (±SD)	37.0 ± 0.4
Hemodialysis	
Ultrafiltration, L (±SD)	-3.8 ± 0.8
Dialysate sodium concentration, mmol/L (±SD)	143.0 ± 3.5
Dialysate temperature, °C (±SD)	36.4 ± 0.5
Pre-dialysis plasma sodium concentration, mmol/L (±SD)	137.8 ± 4.3
Post-dialysis plasma sodium concentration, mmol/L (±SD)	140.8 ± 3.1
Intradialytic sodium gradient (±SD)	5.1 ± 2.7
Intradialytic sodium balance, mmol (±SD)	-323 ± 242
Pre-dialysis plasma creatinine concentration, μmol/L (±SD)	427.2 ± 211.8
Post-dialysis plasma creatinine concentration, μmol/L (±SD)	240.4 ± 119.5
Pre-dialysis plasma osmolality, mmol/kg (±SD)	301.5 ± 13.3
Post-dialysis plasma osmolality, mmol/kg (±SD)	297.3 ± 7.3

SAPS II, Simple Acute Physiology Score; SD, standard deviation.

### Fluid dynamics induced by hemodialysis

**Figure 2:**
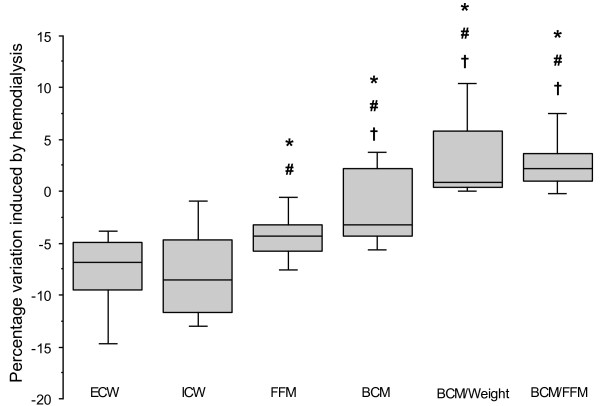
**Box-plots of intradialytic percent variations of extracellular water (ECW), intracellular water (ICW), fat-free mass (FFM), body cell mass (BCM), BCM/weight and BCM/FFM ratios in 31 critically ill patients.** Hemodialysis similarly decreased ECW and ICW, moderately diminished FFM while it did not clinically alter BCM and increased BCM/weight and BCM/FFM ratios. *P <0.001 versus ECW; ^**#**^*P* <0.001 versus ICW; ^**†**^*P* < 0.001 versus FFM.

**Figure 3:**
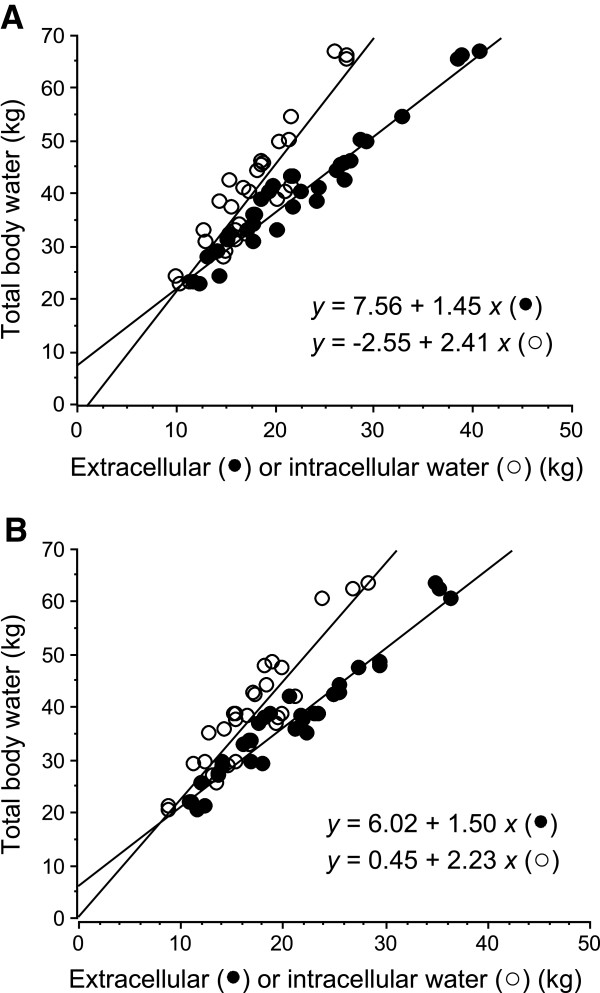
**Pre- (A) and post-dialysis (B) relationships between extracellular water (full circles) or intracellular water (open circles) and total body water in 31 critically ill patients.** The amount of total body water explained by the extracellular water and by the intracellular water was similar in pre-dialysis and in post-dialysis.

**Figure 4:**
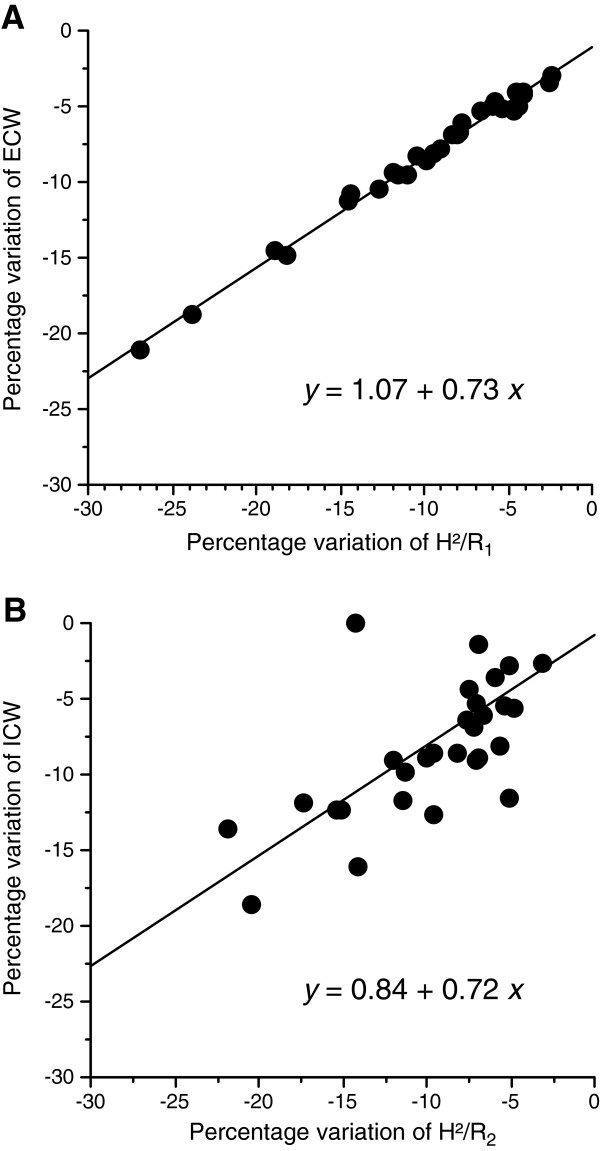
**Relationships between intradialytic percentage variation of tissue resistance measured by bioelectrical impedance at 5 kHz (R**_**1**_**) and percentage variation of extracellular water (ECW) (A), and between percentage variation of tissue resistance measured by bioelectrical impedance at 1 MHz (R**_**2**_**) and percentage variation of intracellular water (ICW) (B) in 31 critically ill patients.** R_1_ and R_2_ were normalized by height^2^ (H^2^). H^2^/R_1_ is a linear function of ECW and H^2^/R_2_ is correlated with fat-free mass and body cell mass in healthy adults.

Tissue resistances measured by BIA at 5 kHz and at 1 MHz significantly increased after hemodialysis (Table 2). As expected, ECW and ICW were significantly diminished by hemodialysis. Intradialytic percentage changes of ECW and of ICW were similar (Figure 2), resulting in a constant ECW/ICW ratio (Table 2). When TBW was considered in relation to the ECW, the amount of TBW explained by the ECW was comparable before and after hemodialysis (adjusted *r*^2^ values, 0.95 (95% confidence interval (CI) = 0.88 to 0.96) and 0.94 (95% CI = 0.87 to 0.96), respectively) (Figure 3A). Similarly, the amount of TBW explained by the ICW was similar before and after hemodialysis (adjusted *r*^2^ values, 0.84 (95% CI = 0.69 to 0.92) and 0.84 (95% CI = 0.68 to 0.92), respectively) (Figure 3B). Intradialytic percentage variations of H^2^/R_1_ and H^2^/R_2_ were -9.49 ± 6.30% and -9.32 ± 4.90%, respectively. As expected, there was a perfect correlation between percentage variation of ECW and percentage variation of H^2^/R_1_ (*r*^*2*^ = 0.99, *r* = 0.99 (95% CI = 0.99 to 0.99); *P* <0.001) whereas the relationship between percentage variation of ICW and percentage variation of H^2^/R_2_ was less (*r*^*2*^ = 0.49, *r* = 0.69 (95% CI = 0.46 to 0.84); *P* <0.001) (Figure 4). The percentage variation of ECW was also more closely correlated with the intradialytic sodium balance (*r*^*2*^ = 0.28, *r* = 0.53 (95% CI = 0.21 to 0.74); *P* = 0.002] than the percentage variation of ICW (*r*^*2*^ = 0.14, *r* = 0.37 (95% CI = 0.02 to 0.64); *P* = 0.03). Patients admitted for cardiogenic shock had significantly higher intradialytic percentage variation of ECW (Table 3) and patients with and without cardiogenic shock at ICU admission were similar for pre-dialysis characteristics (not shown). No other significant relationship was found between intradialytic percentage variation of ECW and the pre-dialysis variables (Table 4). Higher SAPS II at ICU admission was associated with higher percentage variation of ICW (Table 5) whereas the SAPS II at ICU admission was not related with any other pre-dialysis characteristics of the patients (not shown).

**Table 2:** Pre- and post-dialysis bioelectrical measurements, water compartments, body cell mass and fat-free mass for the 31 ICU patients evaluated for body composition

**Measurements**	**Pre-dialysis**	**Post-dialysis**	***P *** **value**
R_1_ (5 Khz), Ω (±SD)	366.7 ± 73.7	407.1 ± 88.3	<0.001
R_2_ (1 Mhz), Ω (±SD)	425.9 ± 94.2	471.9 ± 116.3	<0.001
Height^2^/R_1_, cm^2^/Ω (±SD)	80.1 ± 23.6	72.4 ± 21.1	<0.001
Height^2^/R_2_, cm^2^/Ω (±SD)	69.7 ± 21.5	63.3 ± 20.5	<0.001
Weight, kg (±SD)	68.4 ± 11.2	65.1 ± 10.8	<0.001
ECW, kg (±SD)	22.8 ± 7.8	20.9 ± 7.1	<0.001
ICW, kg (±SD)	17.9 ± 4.4	16.6 ± 4.5	<0.001
ECW/ICW (±SD)	1.26 ± 0.2	1.26 ± 0.2	0.84
BCM, kg (±SD)	24.3 ± 3.8	24.0 ± 3.8	0.61
FFM^a^, kg (±SD)	49.1 ± 10.2	46.8 ± 9.4	0.03
BCM/weight, %	36.2 ± 6.3	37.5 ± 6.9	<0.001
BCM/FFM, %	50.5 ± 6.8	52.1 ± 7.2	<0.001

^a^Including bone mineral. BCM, body cell mass; BIA, bioelectrical impedance analysis; ECW, extracellular water; FFM, fat-free mass; ICW, intracellular water; SD, standard deviation.

**Table 3:** Association between intradialytic percentage changes of intra- and extracellular water compartments and clinical characteristics at ICU admission (number = 31)

	***n***	**Change in ECW, % (±SD)**	**Change in ICW, % (±SD)**
Reason for ICU admission			
Septic shock	16	-7.13 ± 4.26	-6.95 ± 3.66
Cardiogenic shock	7	-12.21 ± 4.99^a^	-10.92 ± 7.52
Neurological failure	5	-5.64 ± 1.36	-4.90 ± 2.7
Postoperative complications	3	-7.28 ± 3.71	-7.28 ± 3.71
Sex			
Male	19	-7.76 ± 4.83	-7.36 ± 5.38
Female	12	-8.03 ± 4.84	-8.33 ± 4.39
Malnutrition at ICU admission			
Present	4	-8.08 ± 4.75	-7.67 ± 5.31
Absent	27	-7.97 ± 4.77	-7.28 ± 5.99

^a^*P* = 0.02 by Kruskal-Wallis test. ECW, extracellular water; ICW, intracellular water; **SD, standard deviation.**

**Table 4:** Relationship between intradialytic percentage change in extracellular water measured by bioelectrical impedance and pre-dialysis variables collected for the 31 patients

	***r***^**2**^	***r***	**95% CI**	***P***
Age, years	0.02	-0.15	-0.48 to 0.21	0.40
SAPS II at ICU admission	0.001	-0.03	-0.38 to 0.32	0.85
Length of ICU stay before measurements, days	0.002	-0.05	-0.30 to 0.40	0.77
Weight, kg	< 0.001	-0.002	0.35 to 0.35	0.98
Height, cm	< 0.001	0.07	-0.28 to 0.41	0.68
Body mass index, kg/cm^2^	< 0.001	0.01	-0.34 to 0.36	0.94
Body temperature, °C	0.10	-0.32	-0.60 to 0.004	0.08
Plasma sodium concentration, mmol/L	0.09	0.31	-0.05 to 0.59	0.09
Plasma creatinine concentration, μmol/L	< 0.001	-0.07	-0.41 to 0.28	0.69
Plasma osmolality, mmol/kg	0.02	-0.16	-0.48 to 0.20	0.39
Ultrafiltration, L	0.05	-0.22	-0.53 to 0.14	0.23
Dialysate temperature, °C	< 0.001	0.01	-0.34 to 0.36	0.94
Dialysate sodium concentration, mmol/L	0.03	0.17	-0.21 to 0.26	0.34

*P* value <0.05 was considered significant, number = 31. CI, confidence interval (95%); SAPS II, Simple Acute Physiology Score.

**Table 5:** Relationship between intradialytic percentage change in intracellular water measured by bioelectrical impedance and pre-dialysis variables collected for the 31 patients

	***r***^**2**^	**r**	**95% CI**	***P***
Age, years	0.05	-0.22	-0.53 to 0.14	0.23
SAPS II at ICU admission	0.18	0.43	0.08 to 0.67	0.01
Length of ICU stay before measurements, days	0.08	0.29	-0.06 to 0.58	0.10
Weight, kg	0.01	0.10	-0.25 to 0.44	0.44
Height, cm	0.002	0.05	-0.30 to 0.40	0.75
Body mass index, kg/cm^2^	0.006	0.08	-0.27 to 0.42	0.64
Body temperature, °C	0.01	-0.12	-0.45 to 0.24	0.51
Plasma sodium concentration, mmol/L	0.04	0.20	-0.16 to 0.53	0.26
Plasma creatinine concentration, μmol/L	0.002	0.05	-0.30 to 0.39	0.78
Plasma osmolality, mmol/kg	0.04	0.21	-0.15 to 0.52	0.26
Ultrafiltration, L	0.001	-0.04	-0.39 to 0.31	0.81
Dialysate temperature, °C	0.07	-0.27	-0.57 to 0.09	0.14
Dialysate sodium concentration, mmol/L	0.02	0.15	-0.21 to 0.48	0.40

*P* value <0.05 was considered significant, number = 31. CI, confidence interval (95%); SAPS II, Simple Acute Physiology Score.

### Body cell mass and fat-free mass dynamics induced by hemodialysis

The intradialytic fall of FFM (-2.24 ± 1.56 kg, -4.43 ± 2.65%) was less pronounced than the decrease of ECW and ICW (Figure 2). Unlike ECW and ICW, intradialytic variation of BCM was clinically irrelevant (-0.38 ± 0.93 kg, -1.56 ± 3.94%) while BCM/weight and BCM/FFM significantly increased during the hemodialysis session (Table 2, Figure 2). Hydration of FFM (TBW/FFM) was 81.9 ± 9.6% before hemodialysis and 79.2 ± 9.2% after dialysis (*P* <0.001). The relationships between intradialytic percentage change of BCM and intradialytic percentage change of H^2^/R_2_ was moderate (*r*^*2*^ = 0.27, *r* = -0.52 (95% CI = -0.73 to -0.20); *P* = 0.002) and no significant relationship was found between intradialytic percentage change of FFM and intradialytic percentage change of H^2^/R_2_ (*r*^*2*^ = 0.04, *r* = 0.21 (95% CI = -0.15 to 0.23); *P* = 0.24).

## Discussion

The results of this study show similar intradialytic variations of ECW, ICW and TBW in anuric ICU patients with fluid overload. Hemodialysis-induced sudden fluid shifts significantly impacted FFM estimation while change in BCM remained clinically negligible. These findings suggest that estimation of BCM should be preferred to FFM when severe hydration disturbances are present. To the best of our knowledge, this is the first study that assessed at bedside BCM and water compartments for evaluating the impact of fluid dynamics on body composition in severely ill patients. However, we were cautious in selecting steady-state patients by using criteria to avoid bias in measurements of water compartments and in BCM calculation, limiting the generalization of our results to other ICU populations.

The consequences of chronic hemodialysis on body composition have been largely investigated [20,35,36]. In patients with chronic renal insufficiency, a hemodialysis session reduced ECW whereas there has been conflicting evidence regarding its impact on BCM and ICW [37-39]. These conflicting findings could be explained by population specific results or by the limitations of single-BIA in the case of fluid overload or fast fluid shifts. Moreover, it has been suggested that patients with chronic kidney dialysis have essentially an increase in ECW while ICU patients with acute renal failure could have global fluid overload [27,40]. Herein, we observed that hemodialysis increased low- and high-frequency resistances, confirming results obtained in patients with chronic kidney failure. Pertinently, increased resistance after fluid drainage in both the low- and high-frequency range is achieved due to higher resistivity of limbs than the trunk which contains the main part of BCM [41].

During chronic hemodialysis, intradialytic changes in ECW or ICW are influenced by various factors, such as sodium balance, the reduction of ECW velocity and the decrease in plasma urea concentration [42]. We established that the amounts of TBW explained by ECW or ICW remained unchanged in the ICU patients, indicating no intradialytic fluid shifts between water compartments. Conversely, substantial fluid movements between ICW and ECW have been reported during chronic hemodialysis [20,27,39,42]. Finally, intercompartmental fluid shifts induced by hemodialysis could explain why moderate correlation between intradialytic sodium balance and variations of ECW was found in the present study.

Among dialysis patients, TBW mainly reflects the muscle mass but not the visceral mass that houses the internal organs (trunk) and the brain [23,42]. While arms and legs contribute to only 4% and 17% of body weight, they contribute to 47% and 50% of whole body resistance [12]. Therefore, multi-frequency BIA is highly sensitive to fluids shifts in arms and legs [41], indicating ECW and ICW variations had little effects on BCM in the present study. Indeed, trunk length is a key determinant of BCM whereas only the leg circumference is taken into account in our calculation of BCM. In ICU patients, ECW and ICW inflation lead to global water redistribution altering FFM which contains TBW [12].

In healthy subjects, ICW is described as a component of BCM. Intriguingly, we established herein that the decrease in ICW was not associated with a similar decrease in BCM following sudden fluid shifts in critically ill patients. Our BCM calculating method has been developed from the Cohn’s model which expresses the BCM as the subtraction of extracellular solids and extracellular fluids from FFM; as a result ICW is not a direct determinant of BCM in our model [12]. This is important for interpreting our results. Indeed, critically ill patients have major water retention in both intra- and extracellular compartments associated with increased capillary and cell membrane permeability due to aggression and inflammatory reactions. Moreover, intercompartmental fluid shifts induced by hemodialysis are dependent on refilling of plasma volume from interstitial spaces and on postdialysis (30 minutes) urea rebound which equilibrates urea concentrations and osmolality across body water compartments [43,44]. Therefore, in critically ill patients, water compartments volumes do not correspond necessarily to anatomic spaces or standard body compartments of normal subjects [44]. In this way, our new model of BCM calculation is widely free from much of the intercompartmental fluid shift occurring in critically ill patients as suggested by the present study.

A limitation of this study is the relatively small sample size; therefore, caution is needed before extrapolating the results beyond the patient population and procedures of hemodialysis. Moreover, it is known that underlying disease impacts the water repartition in the body [45]. This fact probably explains the relationships between ECW shift and patients admitted for cardiogenic shock in the present study. In addition, the severity of the underlying disease at ICU admission could impact ICW shift during hemodialysis as shown herein. Another limitation is the undervaluation of the hydration of lean muscle mass [17] that would explain the weaker correlation between ICW shift and H^2^/R_2_.

## Conclusions

The present findings show that acute hemodialysis-induced fluid dynamics and changes in body composition of ICU patients differ from known data in chronic dialysis patients. Our model of BCM calculation appears to be less driven by sudden massive fluid shifts than FMM, suggesting that BCM calculation at bedside may represent a useful tool for the nutritional assessment of ICU patients when severe hydration disturbances are present. Future work based on the results of the present study will be needed to determine the impact of continuing BCM fluctuations on nutritional assessment of ICU patients.

## Key messages

• Estimation of body composition as FFM is subjected to many variations caused by injury and stress conditions in the ICU. BCM, the metabolically active part of FFM, is reported to be more specifically correlated to changes in nutritional status. Bedside estimation of BCM could help to provide more valuable markers of nutritional status and may promote understanding of metabolic consequences of energy deficit in ICU patients.

• During acute hemodialysis, similar variations of ECW and ICW compartments occur in ICU patients whereas intradialytic variation of BCM is negligible and remains significantly less than FFM, suggesting substantial intercompartmental fluid shifts induced by hemodialysis.

• In the case of sudden and massive fluid shifts, our study suggests that assessment of BCM should be preferred to FFM estimation in ICU patients.

## Abbreviations

BIA: multi-frequency bioelectrical impedance; BCM: body cell mass; BMI: body mass index; CI: confidence interval; ECF: extracellular fluid; ECS: extracellular solids; ECW: extracellular water volume; FFM: fat-free mass; GNa: intradialytic sodium gradient; ICW: intracellular water volume; Mo: bone mineral; R: tissue resistance measured by bioelectrical impedance; REE: resting energy expenditure; SAPS: Simple Acute Physiology Score; SD: standard deviation; TBW: total body water volume; UF: ultrafiltration.

## Competing interests

The authors declare that they have no competing interests.

## Authors’ contributions

SI and MS contributed equally to the manuscript. CF conceived the study, interpreted the data and drafted the manuscript. MS performed measurements and drafted the manuscript. SI interpreted the data and drafted the manuscript. FG performed statistical analyses and drafted the manuscript. CD participated in the design and coordination of the study and drafted and revised the manuscript. CT participated in the design and coordination of the study and drafted and revised the manuscript. All authors read and approved the final manuscript.
